# A Survey on the Use of Cannabidiol (CBD) Isolate, Its Perceived Benefits, and Associated Side Effects Among Subjects With Chronic Pain

**DOI:** 10.7759/cureus.80198

**Published:** 2025-03-07

**Authors:** Austin Huang, Laurence Stolzenberg, Mohammad Usman, Muhammad Awan, Paul Bruner, Gordon MacGregor

**Affiliations:** 1 Neurology, Alabama College of Osteopathic Medicine, Dothan, USA; 2 Radiology, Firelands Regional Medical Center, Sandusky, USA; 3 Psychiatry, Alabama College of Osteopathic Medicine, Dothan, USA; 4 Clinical Sciences, Alabama College of Osteopathic Medicine, Dothan, USA; 5 Family Medicine, Northern Ohio Medical Specialists (NOMS) Healthcare, Sandusky, USA; 6 Pharmacology, Alabama College of Osteopathic Medicine, Dothan, USA

**Keywords:** cannabis use, cbd products, chronic pain management, marijuana use, opioid medication

## Abstract

Introduction: Chronic pain is a pervasive health issue in the United States, affecting up to 50 million adults and contributing to a substantial economic burden. The worsening opioid crisis has increased the need for nonopiate, nonaddictive alternatives to pain management. Cannabidiol (CBD) has recently garnered attention for its potential analgesic and anti-inflammatory properties without addictive or dependence potential. However, there is limited research regarding the efficacy and safety of CBD in the treatment of chronic pain. This study aimed to explore the use of CBD isolate in adults suffering from chronic pain.

Methods: We decided to investigate its perceived effectiveness, dosage, frequency of use, and reported side effects through a publicly available, completely anonymous Qualtrics survey. This survey was posted online and in multiple medical clinics.

Results: The key findings of our anonymous online survey are that the use of cannabinoids is positively associated with decreased chronic pain, even at low dosages (<100 mg). In addition, though some mild side effects are noted, the majority of patients self-report no side effects, and there are no noted severe side effects.

Conclusion: These findings suggest that CBD may serve as a promising alternative to conventional pain management strategies. However, the results warrant further investigation and research into the efficacy and safety of CBD for chronic pain. We believe these data point the way for new and continued avenues of research that can better optimize treatment regimens and help patients with chronic pain.

## Introduction

Chronic pain is a major health concern experienced by a significant portion of the population, making this condition one of the most common reasons adults seek medical care [1]. Chronic pain accounts for approximately 20% of all outpatient visits [2]. It has been estimated that 50 million adults in the United States (US) have experienced chronic pain [1]. Multiple research studies have found a significant decrease in the quality of life of patients due to chronic pain and have called for more effective methods of treatment, as well as promoting an interprofessional team approach [3-5]. However, treatment can be difficult, and one further complicating factor is the impact of the opioid crisis. Chronic pain is also a massive economic issue, as it has been estimated to cost the US economy potentially upward of 600 billion dollars a year, half of which comes from direct medical expenses. As such, any intervention that can reduce chronic pain in a safe manner is desperately needed by vast swathes of the population. Chronic pain can lead not only to a decrease in quality of life but also to a reduced ability to work, reduced salaries or lost wages, worsening of chronic disease, and psychiatric disorders [3].

The widespread use of opioids to treat acute and chronic pain has contributed to more than 564,000 deaths from overdoses involving any opioid from 1999 to 2020 [6]. In 2022 alone, the Centers for Disease Control estimated that there were approximately 80,000 deaths as a result of an opioid overdose. Significant measures have been taken to address the opioid epidemic, such as the limitation on the duration of opioid therapy (i.e., shortening the number of days’ supply to decrease the risk of drug dependence) [7]. However, these major restrictions in the prescription of opioids for the management of chronic pain have resulted in many chronic pain patients struggling to manage their conditions sufficiently. As clinicians, we must investigate alternative treatments for chronic pain to mitigate the negative impacts of opioids. Cannabidiol (CBD) is a potential alternative treatment that may play a vital role in this discussion.

CBD is the second most abundant phytocannabinoid in *Cannabis sativa* Linnaeus, and it has gained increased attention in both the research literature and the public eye for a variety of reasons. It is especially intriguing from a medical perspective for its use in the management of various conditions (e.g., approved for epilepsy, off-label for anxiety, and off-label for dystonia) [8]. CBD, as well as its counterpart Δ9-tetrahydrocannabinol (THC), acts via the endocannabinoid system in the brain. THC is more famously known for being the substance in marijuana that is responsible for the intoxicating effects. However, unlike THC, CBD does not have any intoxicating activity, addictive properties, or dependence potential. It provides analgesic and anti-inflammatory mechanisms by inhibiting cyclooxygenase and lipoxygenase [9].

While there have been some studies into CBD as a whole (e.g., cross-sectional, cohort), the safety and efficacy of CBD have not been well established, as very few studies have set out to investigate this particular facet. While there has been a substantial increase in research investigating the uses of CBD in the recent past, there remains a substantial gap in the literature. Notably, one of the biggest gaps in the literature has been the fact that there is very little understanding of what the general public thinks about the potential medical benefits of CBD. Considering the theoretical rationale for the efficacy of CBD, as well as prior research suggesting pain relief with CBD, the research team sets out to further explore this topic and add to the literature with the aim of asking chronic pain patients who use CBD for pain relief about their perceived benefits and side effects of CBD.

## Materials and methods

In the current study, we aimed to collect insight into the use of CBD isolate in adults with chronic pain. We sought to investigate specifically not only its use but also its perceived effectiveness and side effects via a validated 16-item Qualtrics survey. Before the current study, multiple physicians practicing in Alabama and Florida were recruited to direct their patients to fill out the survey. Recruitment flyers were provided to the participating physicians to post at their clinics, and handouts were given to visiting patients who met the study’s inclusion criteria. Additionally, with permission, these flyers were posted in various healthcare facilities in Alabama and Florida. The survey was posted online via various social media platforms, including Reddit, Twitter, and Facebook. The survey was made available to patients and the population from May 2022 through September 2022.

The initial study population included anyone with internet access, who was over the age of 21, and who suffered from chronic pain. These criteria were chosen to allow for the widest possible participant pool. A total of 216 responses were received, which were then filtered to exclude participants who completed the entire survey in less than 40 seconds and those who had not answered all the questions. Furthermore, entries were filtered if no age, age under 21, or if no dose of CBD was specified by the respondent. Upon applying the data clean-up filter, a total of 121 responses remained. All statistical analyses of the data collected during the survey were performed using Microsoft Excel (Microsoft Corporation, Redmond, WA) or GraphPad Prism (Dotmatics, Boston, MA).

We collected several variables during the survey. The first data we collected were the necessary background information, such as the time it took to complete the survey, the age of the respondents, and their self-reported gender (male, female, prefer not to disclose, self-disclose with a text box). Survey participants were then asked to provide the cause of their chronic pain by selecting a diagnosis from a predefined list of common causes of chronic pain, with the alternative option of adding a different cause of chronic pain in the “Other” section.

The respondents were questioned on the number of years they had been using CBD for chronic pain; respondents who had been using CBD for less than a year were asked to enter one year. Next, subjects provided the frequency of their CBD use, specifically the number of times they used it daily. Participants were also questioned on their dose of CBD at the time of the questionnaire.

Subjects were asked to provide their baseline level of chronic pain before CBD use using a numerical rating scale (NRS) from 0 to 10, where 0 was no pain at all and 10 was the worst pain they had ever felt. In a separate question, participants self-reported the reduction in their chronic pain after using CBD. The reduction in chronic pain was also measured using an NRS from 0 to 10; in this case, 0 was no reduction in pain at all, and 10 signified that the pain had completely resolved. To convert the reduction in pain to adjusted levels of chronic pain after CBD, the following formula was produced: adjusted pain level = A - (B/10) * A, where A is the baseline chronic pain value and B is a self-reported reduction in chronic pain value after CBD. For example, if the baseline level pain of a subject was 5 and the reported reduction in pain was 6 out of 10, the equation would be as follows: 5 - (6/10) * 5 = 2. Hence, the adjusted pain level after CBD is a 2/10 for that subject.

Finally, survey participants were asked to report side effects from CBD use, with the ability of multiple side effects to be selected, and a text box allowing additional side effects outside the most common ones that were listed.

IRB approval and informed consent

The research protocol was reviewed by the Alabama College of Osteopathic Medicine Institutional Review Board (IRB) and was determined to be exempt from further IRB oversight (HS220517-E-1). Informed consent was collected on the first page of the survey.

Inclusion and exclusion criteria

The included population was men and women, 21 years old and above, who self-identified as having chronic pain. We defined chronic pain as pain lasting six months or longer.

The survey included several initial screening questions to determine if the participant fulfilled the inclusion criteria. If it was determined that the participant did not fulfill the requirements or was excluded, the survey automatically closed. The survey was completely anonymous and confidential, and no identifying information was collected. The survey asked questions regarding minimal personal health information and level of pain using the NRS.

## Results

Survey response rate

The survey received a total of 216 responses. After filtering out the results of those who answered the entire survey in less than 40 seconds, those who had not answered all questions, and those who did not mark a response to how much pain they had, we were left with 56% (n = 121) of our initial responses.

Demographic characteristics of survey respondents

The average age of respondents was 37 years old (range 21-67), with 61.2% (n = 74) being male, 33.1% (n = 40) being female, 1.6% (n = 2) who self-disclosed as gender fluid or other, and 4.1% (n = 5) who preferred not to disclose (Table 1).

**Table 1:** Demographic data CBD: cannabidiol

Variables	Category	Frequency	Percent
Gender	Male	74	61.2
	Female	40	33.1
	Prefer not to disclose	5	4.1
	Self-disclosed	2	1.6
Age	21-30	45	37.2
	31-40	36	29.8
	41-50	20	16.5
	51+	20	16.5
Using CBD for chronic pain	Yes	121	100

Self-reported duration of chronic pain

Subjects were asked about the duration of their chronic pain. A total of 100 participants responded that their chronic pain had lasted for 24 months or longer, while 21 respondents reported a duration of chronic pain of 23 months or less.

Predominant causes of chronic pain in participants

Survey participants were asked to provide the cause of their chronic pain. Table 2 includes the categories and frequencies of causes of chronic pain in survey participants. The leading causes of chronic pain were arthritis in 15.7% (n = 19) of subjects, disk herniation in 14.9% (n = 18), fibromyalgia in 7.4% (n = 9), headache or migraine in 6.6% (n = 8), and neuropathy in 6.6% (n = 8). Additional causes of chronic pain provided by participants in the “Other” section included spinal stenosis, injuries from car accidents or sports, and polycystic kidney disease, among others. Multiple options could be selected, so the total number of responses was greater than the number of survey participants (Table 2).

**Table 2:** Causes of chronic pain The main causes of chronic pain, frequency, and percentage of total respondents

Cause of pain	Frequency	Percent
Arthritis	19	15.7
Disk herniation	18	14.9
Fibromyalgia	9	7.4
Headache or migraine	8	6.6
Neuropathy	8	6.6
Irritable bowel syndrome	6	5.0
Vertebral fracture	3	2.5
Sciatica	2	1.6
Postsurgical pain	2	1.6
Psychosomatic	2	1.6
Ankylosing spondylitis	1	0.8
Endometriosis	1	0.8
Chemotherapy causing bone pain	1	0.8
Lupus	1	0.8
Multiple sclerosis	1	0.8
Behcet's disease	1	0.8
Other	44	36.4

Length of time of CBD use by survey participants

**Figure 1:**
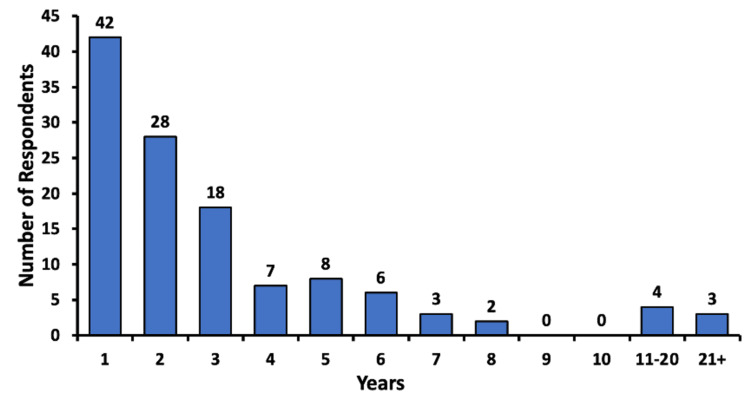
Self-reported number of years of cannabidiol use For under one year of use, respondents were requested to enter one year

The respondents were asked about the number of years they had been using CBD for chronic pain. A total of 34.7% (n = 42) of respondents had used CBD for a year or less than a year at the time of the survey; 23.1% (n = 28) of participants had used CBD for two years, and 14.9% (n = 18) of participants for three years; and 3.3% (n = 4) of participants used CBD for 11-20 years, and 2.5% (n = 3) of participants for over 21 years (Figure 1).

Frequency and dose of CBD use by participants

**Figure 2:**
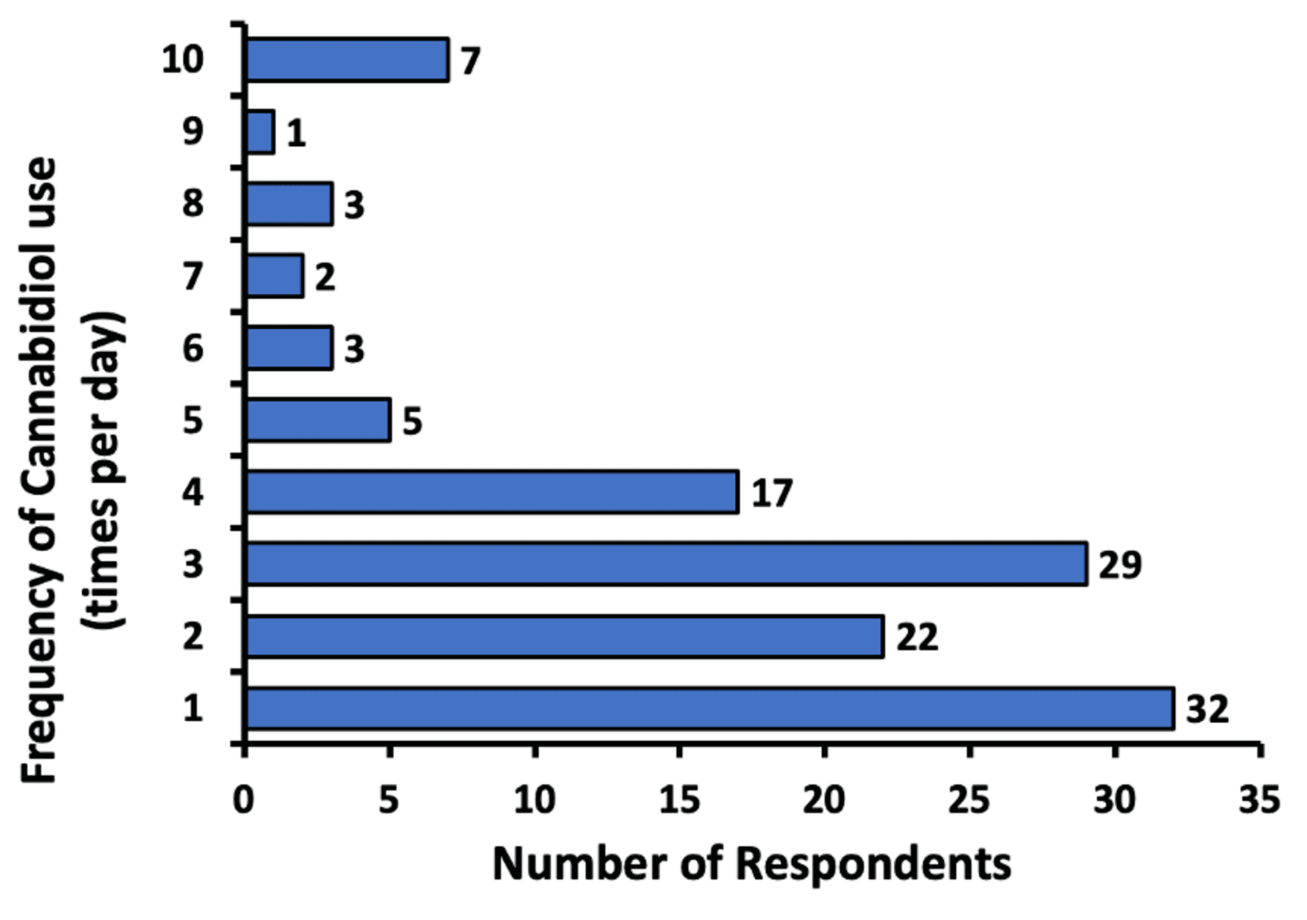
Frequency of cannabidiol use (times per day) Respondents were able to enter how many times a day they were using cannabidiol

Subjects were asked about the frequency and dosage of their CBD use. A total of 26.4% (n = 32) of participants used CBD once a day, followed by 24.0% (n = 29) using CBD three times a day, and 18.2% (n = 22) twice a day. The maximum frequency of CBD use reported by participants was 10 times per day in 5.8% (n = 7) of subjects (Figure 2).

**Figure 3:**
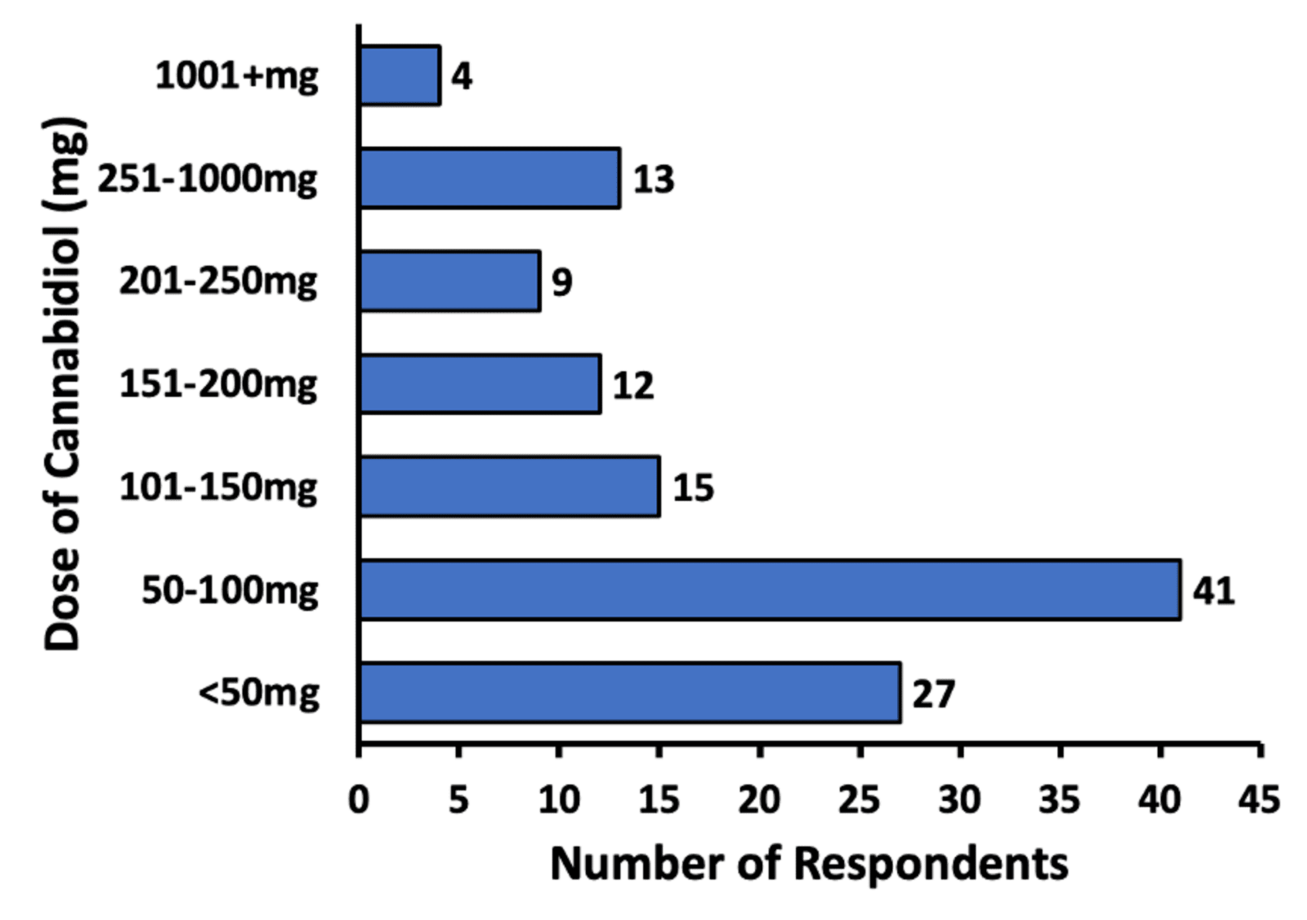
Dose of cannabidiol (milligrams) used by subjects Respondents were able to enter how much cannabidiol they were using daily in milligrams

Regarding CBD dosages, 33.9% (n = 41) of respondents reported a dose of between 50 and 100 mg of CBD, 22.3% (n = 27) of subjects used a dose of less than 50 mg, and 3.3% (n = 4) of subjects used a dose of >1,000 mg. The ranges of CBD dosage and the number of participants who reported using a dose within each range are depicted in Figure 3.

Self-reported reduction of chronic pain after CBD use

**Figure 4:**
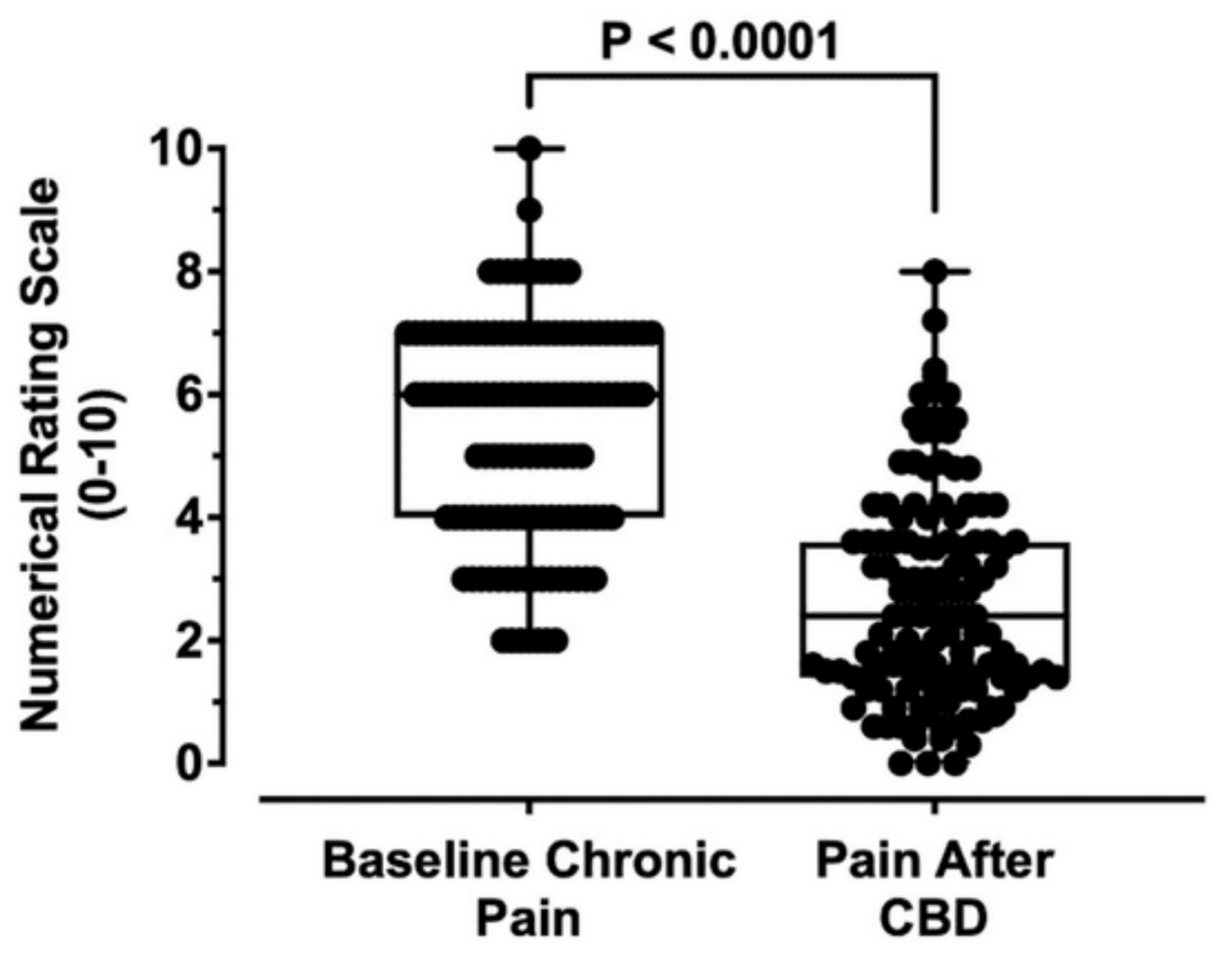
Comparison of baseline chronic pain and calculated chronic pain after CBD Baseline chronic pain rating was 5.4 ± 1.8 (range 2-10, n = 121) and decreased to 2.6 ± 1.7 (range 0-8, n = 121) at p < 0.0001 (Mann-Whitney U test). The bars represent the range of the data, the horizontal solid line represents the median, and the open box represents the interquartile range (Q1-Q3) CBD: cannabidiol

Subjects were asked to provide their baseline level of chronic pain before CBD use on an NRS of 0-10 (Figure 4, left). The most commonly reported chronic pain levels among participants were 6/10 (n = 25) and 7/10 (n = 28). In total, 64.5% (n = 78) of subjects had a baseline chronic pain greater than or equal to 5. Participants were then asked to self-report the reduction in chronic pain after using CBD on the NRS from 0 to 10. The baseline pain rating was adjusted by applying the reduction in pain after CBD using the formula mentioned in the Methods section to all baseline pain values (Figure 4, right). The average baseline level of chronic pain across participants before CBD was 5.4 ± 1.8, which decreased to 2.6 ± 1.7 (p < 0.0001, n = 121) after CBD, which is a decrease of 2.8 ± 1.7 on the NRS. Improvement was seen in 98.3% (n = 119) of subjects, while 1.7% (n = 2) of subjects reported no improvement at all after CBD. Most participants demonstrated an adjusted pain level after CBD of less than or equal to 3/10 (n = 76). Three subjects self-reported complete resolution of their baseline chronic pain after CBD (Figure 4).

Participants’ experience of side effects from CBD use

**Figure 5:**
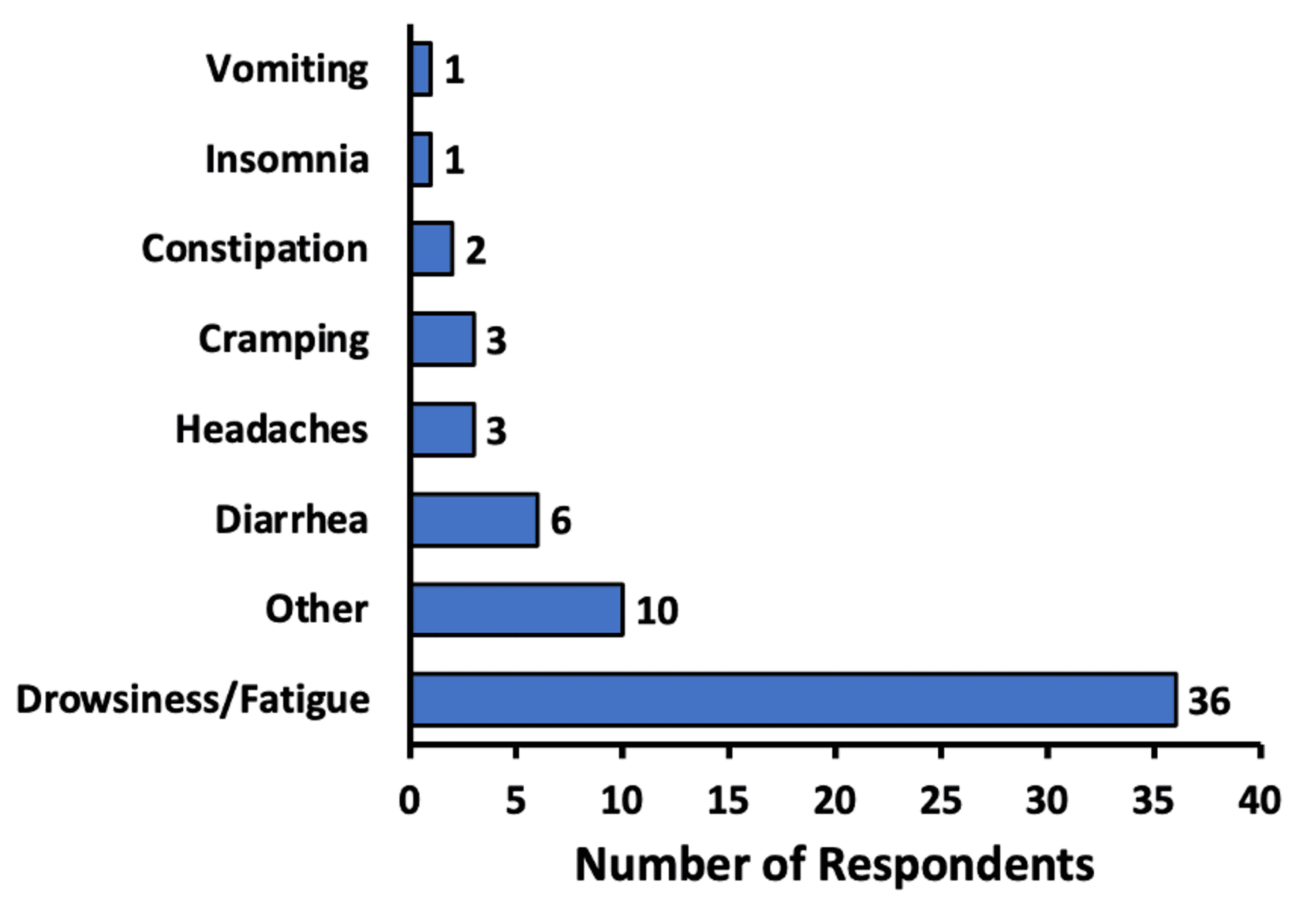
Side effects from cannabidiol use The main side effects for respondents using cannabidiol are listed. Respondents were able to enter what kinds of side effects they experienced from using cannabidiol

Participants were questioned on potential side effects they may have experienced as a result of CBD. Multiple side effects could be selected; hence, the total amount of responses was greater than the number of participants. A majority of participants, 55.4% (n = 67), experienced no side effects at all. Of those who did experience side effects from CBD, 29.8% (n = 36) reported drowsiness and fatigue, followed by diarrhea in 5.0% (n = 6) of respondents, and headaches and cramping in 2.5% (n = 3) of respondents each. Among the “Other” side effects reported, nausea was the most common, seen in 1.6% (n = 2) of respondents. No severe side effects from CBD use were reported (Figure 5).

## Discussion

The current study analyzes the use of CBD isolate in people with chronic pain. Specifically, data from participants regarding dosage and frequency, subjective efficacy in pain relief, and side effects of CBD treatment were evaluated. Our findings indicate that participants utilize CBD for a variety of chronic pain (Table 2). Arthritis, disk herniation, and fibromyalgia were the leading causes of chronic pain reported by respondents. However, these together only represented a little over a third of survey participants, with the remaining participants reporting up to 20 other different causes of chronic pain. These findings indicate that CBD usage as a treatment for chronic pain is not restricted to the pain management of a few specific causes.

The dosage and schedule of CBD usage were analyzed to understand the effectiveness and convenience of CBD in pain management. Nearly 69% of respondents reported using CBD between one and three times daily (Figure 2). Hence, it may be inferred that a majority of subjects are able to feel a reduction in chronic pain by administering CBD three or fewer times per day and that administering CBD for pain does not serve as a burden in the schedule of most people. Moreover, 56.2% of respondents found relief with 100 mg or less of CBD, with only a few needing to increase the dose to above 1,000 mg (Figure 3). These data suggest that CBD may begin to show efficacy in treating chronic pain at smaller doses for most people.

CBD has demonstrated efficacy in the treatment of chronic pain in study respondents, regardless of the cause. Over 98% of participants self-reported an improvement to some extent of their chronic pain, ranging from a minimal reduction in pain (i.e., 1/10), to a complete resolution of chronic pain (i.e., 10/10), through CBD treatment (Figure 4). This effectiveness of CBD in treating various causes of chronic pain may be attributed to its multiple mechanisms of action. CBD provides analgesic and anti-inflammatory effects by inhibiting cyclooxygenase and lipoxygenase, inhibiting the production of leukotrienes in polymorphonuclear cells, acting as an allosteric modulator of the mu and delta opioid receptors, and partially activating the serotonin 5-HT1A receptor [9].

In a recent study on the effect of CBD as a treatment for pain in arthritis, an absolute reduction of 2.6 ± 2.1 in pain was reported by the subjects on the 0-10 NRS [10]. This is similar to the 2.8 ± 1.7 decrease in the NRS (0-10) we observed in our study on chronic pain. This is important, as a reduction of approximately 2 points in a 0-10 NRS represents a clinically important difference [11]. In a separate study of chronic musculoskeletal pain, an improvement after treatment considered “much better” was associated with a decrease of at least 2 points on an NRS [12]. Taken together, the subject-reported decrease of 2.8 in the pain NRS that we observed in this study of CBD likely represents a clinically significant decrease in pain in research subjects in this study.

Nonetheless, two subjects with similar causes of chronic pain (e.g., arthritis) as the rest of the respondents reported no reduction in their pain at all following CBD use. This indicates the need for further research to understand why the treatment did not reduce pain in these people.

In terms of adverse effects of treatment, 55.4% of participants experienced no side effects at all with CBD administration. In those who did experience side effects from CBD, nearly a third reported drowsiness and fatigue (Figure 5). Diarrhea, constipation, headache, and cramps were among other uncommon side effects disclosed. Ultimately, no severe side effects from CBD use were reported from any of the respondents.

It is also important to address other similar studies attempting to qualify and quantify the effects of CBD on chronic pain. One study by Capano et al. [13] found that, of the 97 people in their study with chronic pain and opioid use, CBD use resulted in a significant reduction of pain, reduction of opioid use, and an improved quality of life. Conversely, certain papers studying the effect of CBD on older adults had less promising, if somewhat inconsistent, results. One study by Moore et al. [14] showed no benefit over placebo for doses between 6 and 1,600 mg, from a single dose to 12 weeks. They also reported incidences of serious adverse effects, including hepatotoxicity. While there are numerous papers on this issue, many of them seem to reuse the same data and have inconsistent results. We believe our data, obtained anonymously to reduce reporting and other forms of bias, bring valuable additional information to this topic. It is obvious, though, that this subject would benefit from a large meta-analysis or literature review in the future to obtain more conclusive evidence for clinicians.

Limitations

It is clear that there is considerably more research needed into the utilization of CBD with respect to controlling chronic pain. The current study has two main limitations. Offering the survey via social media platforms restricted participation to only those individuals with internet access and with sufficient understanding of computers to access it. Offering the survey in paper and pencil format, or verbally to consenting patients, would increase the sample size and potentially provide a more robust picture of CBD usage for the management of chronic pain. Additionally, participants were asked to report perceived side effects. Future investigations on the utilization of CBD for chronic pain management perhaps should include physician reports or patient records to validate the self-reported side effects. Comparing reports/records to self-reported side effects could provide a clearer understanding of the positives and negatives of using CBD as an alternative to opioids for the treatment of chronic pain.

## Conclusions

The findings from the current project indicate that a majority of participants believe their chronic pain has improved with the usage of a CBD supplement. Most subjects used CBD between one and three times a day, with many finding relief with a dose of 100 mg or less. Furthermore, most respondents experienced either mild side effects or no side effects at all. Altogether, these findings may be comforting to individuals concerned about taking pain medication too frequently, at high doses, or about its associated adverse effects. While our research is certainly not exhaustive, it is a clear indication that the possibility of great benefit of CBD treatment exists in treating chronic pain. As such, additional research is warranted to explore this topic further.
